# Nanostructured Tungsten Oxide Composite for High-Performance Gas Sensors

**DOI:** 10.3390/s151027035

**Published:** 2015-10-23

**Authors:** Siyuan Feng-Chen, Ali Aldalbahi, Peter Xianping Feng

**Affiliations:** 1 Escuela Secundaria de la Universidad de Puerto Rico, San Juan, PR 00936, USA; E-Mail: sfengpr@gmail.com; 2 Department of Chemistry, King Saud University, Riyadh 11451, Saudi Arabia; E-Mail: aaldalbahi@ksu.edu.sa; 3 Department of Physics, University of Puerto Rico, San Juan, PR 00936, USA

**Keywords:** tungsten oxide composite nanowires, gas sensor, sensitivity, response

## Abstract

We report the results of composite tungsten oxide nanowires-based gas sensors. The morphologic surface, crystallographic structures, and chemical compositions of the obtained nanowires have been investigated using scanning electron microscopy (SEM), X-ray diffraction (XRD), and Raman scattering, respectively. The experimental measurements reveal that each wire consists of crystalline nanoparticles with an average diameter of less than 250 nm. By using the synthesized nanowires, highly sensitive prototypic gas sensors have been designed and fabricated. The dependence of the sensitivity of tungsten oxide nanowires to the methane and hydrogen gases as a function of time has been obtained. Various sensing parameters such as sensitivity, response time, stability, and repeatability were investigated in order to reveal the sensing ability.

## 1. Introduction

Oxide semiconductor films exhibit excellent properties for sensor devices [1,2,3,4,5]. There is a strong interest in the development of lightweight gas sensors capable of low ppm range sensitivity and extended operation at low-power levels. Recent experimental results demonstrate that the gas-sensing process is strongly related to surface reactions. Different metal oxide-based materials have different reaction (selectivity) activations to the target gases. Consequently, they have a potential for detecting various gases.

When considering the influence factors on gas-sensing properties of metal oxides, it is necessary to reveal their sensing mechanism. The fundamental mechanisms that cause a gas response are still controversial, but are thought to be essential to the trapping of electrons at adsorbed molecules that induces band bending, resulting in a change in conductivity. Moreover, the reaction of species with reducing gases or a competitive adsorption and replacement of the adsorbed species by other molecules decreases and can reverse the band bending, resulting in the variation of conductivity [6,7].

A variety of techniques for the deposition of oxide semiconductors have been developed [8,9,10,11]. In general, composite metal oxide sensors exhibited significantly higher sensitivity than conventional sensors constructed solely from one material when tested under identical experimental conditions [12].

Nanostructures have been emphasized in the fabrication of gas-sensitive sensors [13,14]. This is in part due to their large surface/volume ratio. For example, Zeng used nano-coral-like porous crystalline WO_3_ film with a grain size of 9.3 nm for a gas sensor detecting NO_2_ at a low operating temperature of 150 °C [15]. Lee fabricated WO_3_ a nano-nodule-decorated carbon nanofiber (CNF)-based gas sensor [16]. It was found that the sensitivity of the hybrid CNF gas sensors increased with the decreasing diameter of the CNFs; the minimum detectable level (MDL) was 1 ppm of NO_2_ gas. Recently, Warmer studied the temperature-dependent sensing properties of metal-oxide semiconductor gas sensors based on SnO_2_ and WO_3_, to measure different target gases [17]. However, the requirement of a heater results in a large and complicated structure, causing some existing sensors to become limited either in their range of application or their sensitivity. George Fine and Jin Huang provided an overview of important contributions and recent advances for the use of metal oxide semiconductor sensors for the detection of a variety of gases, respectively [18,19]. The nature of the gas response and how it is fundamentally linked to the surface structure was explored. Izadyar theoretically studied cyclic nanostructures of tungsten oxide as a sensing material for gas sensors [20].

Based on these achievements above, the present paper focuses on designing and developing simple, low-cost, room-temperature gas sensors based on tungsten oxide composite nanowires. The nanowires were straightly deposited onto a pair of electrodes. No post-growth processing was needed. We directly used as-synthesized samples to pursue the gas-sensing test. No sample destruction had to be operated. This is important for the commercial usage of gas-sensing device manufacturing. The sensing behaviors of the nanowire arrays to different gases at different temperatures have been examined. Response time and recovery time down to few seconds have obtained at the target gas concentration of 10 ppm, whereas at 2 ppm, the response time becomes slow up to 1 min. Furthermore, it was found that the fabricated sensor responded to the methane gas by increasing its resistance, whereas it responded to the hydrogen gas by decreasing its resistance.

## 2. Experimental Setup

The nanostructured tungsten oxide materials were synthesized using a simple hot-filament Chemical Vapor Deposition (CVD) technique. The details of the process are described elsewhere in our previous publications [10,21]. The tungsten filament acted as a precursor for tungsten oxide, and no catalyst or other tungsten-containing compound precursor was used. Both AlN and Al_2_O_3_ ceramic substrates were used. Prior to the experiments, the substrates were ultrasonically washed in a methanol solution for 5 min, and dried with helium. After placing the substrate, the chamber was pumped down to 2.0 × 10^−5^ Torr, and then fed with the Ar gas (99%) and oxygen gas (1%) to 300 mTorr pressure. During deposition, the gas inside the chamber was in a static state. The distance between the hot filament and the substrate remained unchanged. The substrate temperature was controlled by adjusting the electrical current on the hot filament, which is different from our previous experiments where the substrate temperature is controlled by an additional heater under the substrate. Consequently, the present experimental set up was very simple.

## 3. Characterizations of the Tungsten Oxide Composite Nanowires

**Figure 1:**
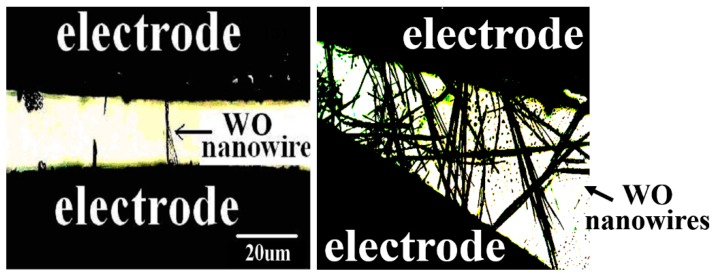
Microscope photograph of the architecture of the prototypical sensor where the nanowires are directly deposited onto a pair of electrodes.

Traditionally, synthesized tungsten oxide nanowires on a ceramic substrate would be scratched onto the metal fringes to form a simple gas sensor. Preliminary experiments were conducted, but it was found that the electrical contacts between the nanowires and the metal fringe electrode arrays were poor, causing low sensitivity in the obtained sensor. Therefore, in the present paper, the doped tungsten oxide composite nanowires are directly deposited onto a pair of metal electrodes as shown in Figure 1.

The sensor is comprised of a pair of electrodes, nanostructured metal oxide semiconducting wires, a heater, a power supply, and an electrical meter. We fabricated both single-wire- and multi-wire-based gas sensors as shown in Figure 1. No obvious differences were found from the preliminary characterization of two gas sensors. Therefore, the focus of the paper is on the multi-wire-based gas sensors. We used metal platinum as electrodes prepared by using the sputtering deposition technique for the sensor. The thickness of the electrode is up to 3 µm.

**Figure 2:**
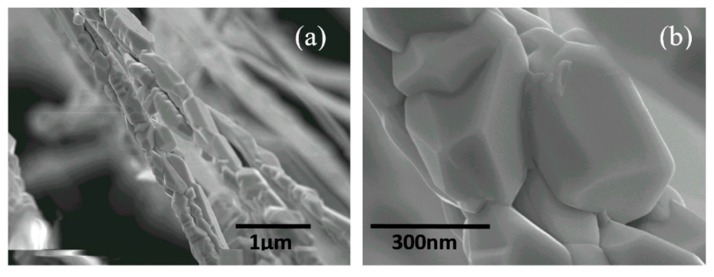
(**a**) Typical SEM images of tungsten oxide composite nanowires, and (**b**) magnified SEM images of selected areas.

Figure 2 shows typical SEM images of the tungsten oxide nanowires at different magnifications. Each as-grown wire normally consists of a large amount of nanoparticles. The well-shaped edge of each particle is clearly visible as shown in Figure 2a, indicating a crystalline structure. The average size of each particle is 250 nm. The average diameter of the wire is around 500 nm, and the average length of the nanowire is up to 1 mm.

**Figure 3:**
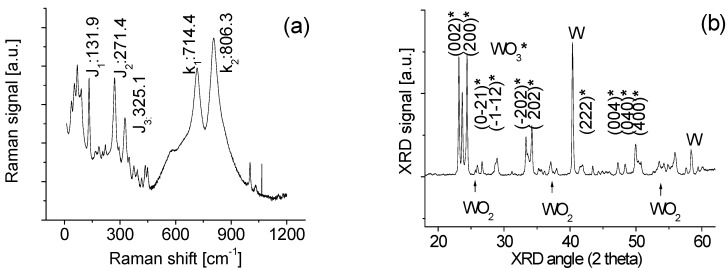
(**a**) Raman; and (**b**) XRD of tungsten oxide composite nanowires.

Raman scattering spectrum of the nanowires was obtained at room temperature by using a triple monochomator (ISA J-Y Model T64000, Louisville, KY, USA) with an excitation wavelength of 514 nm (Ar^+^ ion laser). The samples were scanned from 10 cm^−1^ to 1200 cm^−1^ as shown in Figure 3a. Several peaks marked with J and k in the Raman spectrum were identified. In general, the bands situated at around 700 and 800 cm^−1^ can be assigned to W-O stretching modes, whereas the bands situated at around 130 and 270 cm^−1^ are associated to W-O bending modes of monoclinic WO_3_ [22]. It was also reported that J bands at 270 and 330 cm^−1^ can also be assigned to W-O bending modes of monoclinic WO_3_ [21,23]. In our case, J_1_, J_2_ and J_3_ bands in Raman spectra of all our tungsten oxide samples always coexist. Therefore, the present three bands have been assigned to J bands associated with W-O bending modes of monoclinic WO_3_.

No obvious shifts of the spectral k bands of nanowires were observed after the comparison of spectra of the bulk WO material, indicating that the particles inside the nanowires have less stress. However, the variations of content of WO_2_ and W in the nanowire composite unavoidably change the crystal symmetry of WO_3_ that is qualitatively associated with the increasing width of the band. This is confirmed with X-ray Diffraction (XRD) data.

XRD measurements were conducted to characterize the crystalline structure as shown in Figure 3b. WO_2_, WO_3_ and W peaks in XRD spectrum of the nanowires have been identified based on previous literature [21,23]. The sample exhibits mixed states including WO_2_, WO_3_ and W. The poly-crystalline WO_3_ and metal W dominate the composite of the sample. This is because the intensities of the XRD peaks of W and WO_3_ are much stronger than that of the WO_2_ spectral lines.

## 4. The Results of Fabrication and Characterization of Tungsten Oxide Composite Nanowires-Based Gas Sensors

**Figure 4:**
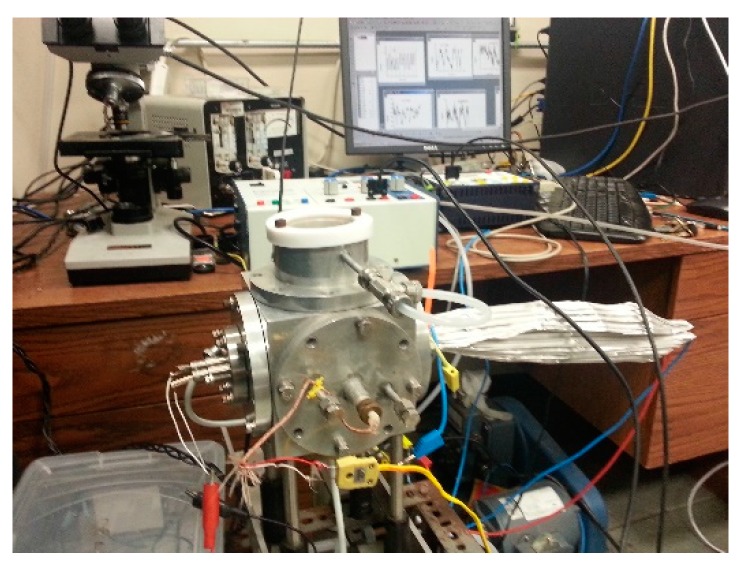
Photograph of experimental set up for characterization of newly fabricated gas sensors.

The prototypic gas sensor is fabricated based on electrodes, nanowires (R_s_), a precise resistor (R_P_), a power supply (V_p_), and an electrical meter (V_m_) connected in series to form a resistance, current, and voltage (R-I-V) electrical circuit. The variation of the electrical current is caused by incoming external molecules of targeted gas that change the nanowire conductivity. From the measurement of variation of voltage (V_m_) across the precise resistor R_P_ = 8.2 kΩ, the variation of the conductivity of the sensor can be immediately obtained by R_s_ = (V_p_ − V_m_)R_p_/V_m_. Necessary calibrations of the sensor conducted at the characterization chamber are shown in Figure 4. It includes a plasma beam source for the treatment of the surface of the sensor device, the mass flow meter (Model FM-360, Tylan Corporation), a precise pressure gauge, a mass spectrometer, *etc.*, for different experimental purposes.

An additional tank was also used to mix the target gas (methane or hydrogen) with nitrogen gas, and two Omega mass flow controllers (one for the target gas and other one for the nitrogen gas) were installed to control target gas concentrations. Because of local high-humidity weather and because we did not desire such a humidity effect on the present sensor, we did not mix the target gas with air. A detailed discussion of the humidity effect can be found in our previous papers [24,25].

The characterizations of the properties of the fabricated sensors include the sensitivity/resistance (R_s_), response time (t_resp_), and recovery time (t_rec_). Repeatability and stability are the most important parameters for a gas sensor. The gas response (S) is defined by directly using the impedance of the sensing material in the fabricated sensor. We did not use a traditional definition of (R_gas_ − R_nitrogen_)/R_nitrogen_ for the response because it could have yielded a negative value for the response when R_gas_ is less than R_nitrogen_.

**Figure 5:**
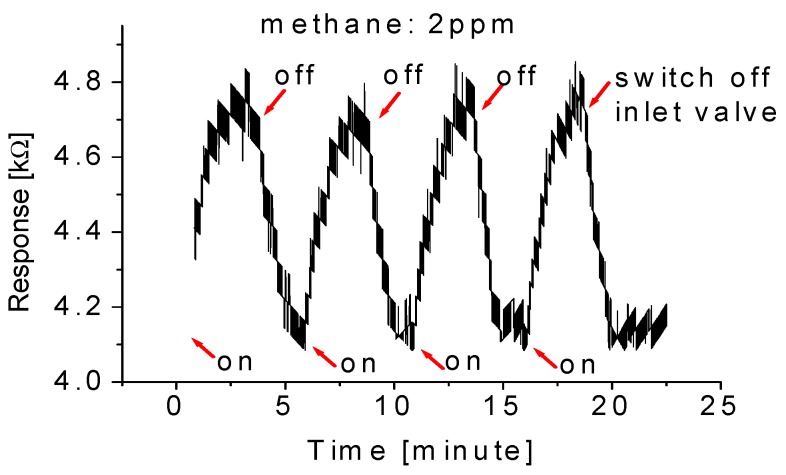
Typical response at room temperature when the nanowire array-based gas sensor is cycled between the “switch-on” and “switch-off” of exposure to the methane gas with a gas concentration of 2 ppm.

Figure 5 shows the typical response at room temperature when the nanowire array-based gas sensor is cycled between the “switch-on” and “switch-off” of exposure to the methane gas. The concentration of the gas molecule is 2 ppm. The sensor has good features in repeatability and stability. It responded to the methane gas by increasing its resistance. The changes in conductivity/resistance can be attributed to the adsorption of methane molecules. Since the shift of the spectral line is observed, it is expected that the adsorption type of methane molecules is chemisorption.

The response and recovery times of the sensors are about 1.5 min and 1 min, respectively. The definition is based on the time duration to reach 90% of the full response of the sensor. It is found that the response-recovery time depends on the nature of the samples, gas concentration, and operating temperature of sensors. In the case where the targeted gas is of low concentration, the time response and recovery are delayed. This can be explained by the fact that there are not enough molecules participating in the reaction with the nanoparticles, and therefore more time is needed for reaching the balance of the reaction between the particle and the targeted molecular gas. In contrast, for the high concentration case, far less time is needed to reach the equilibrium of the reaction. Consequently, short response time can be obtained.

**Figure 6:**
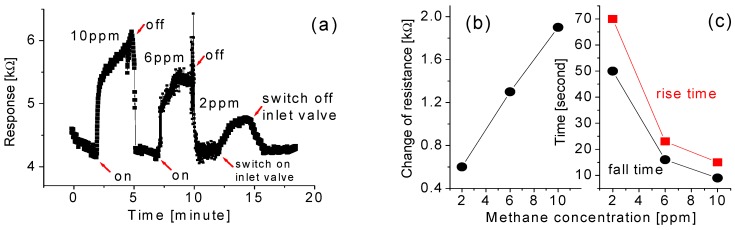
(**a**) The response of the fabricated gas sensor tested at the methane concentrations of 10 ppm, 6 ppm, and 2 ppm, respectively, at room temperature; (**b**) output change (or resistance change); and (**c**) the rise time and fall time as a function of the methane concentrations.

Figure 6a depicts the response of the fabricated gas sensor tested at the methane concentrations of 10 ppm, 6 ppm, and 2 ppm, respectively. The measurements were performed at room temperature. To test the sensor, it is first exposed to 10 ppm methane for 180 s and then the valve was switched off for 120 s. This was then repeated every 5 min with the concentration decreased in steps of 4 ppm for each cycle. An obvious change of electrical resistance of the nanowires was found upon exposure to the methane gas. The variation of resistance was around 1.9 kΩ at 10 ppm of the target gas, 1.3 kΩ at 6 ppm, and 0.6 kΩ at 2 ppm. This possibly indicates that the output change (or resistance change) of the prototypical sensor has an almost linear relationship with the methane concentration within a small area ranging from 2 ppm to 10 ppm. However, we are not able to conclude the trend of the resistance change as a function of the concentration of methane from the limited data.

It is also found that the recovery time is much shorter in the case of high concentration than that of low concentration. For example, in the 10 ppm case, the recovery time is only a few seconds as shown in Figure 6c, which is much shorter than the recovery time of the conventional sensors which are usually about 100 s and more than 500 s, respectively. It is relatively difficult to precisely identify the rise time because the profile can be divided into several areas. According to Figure 6a, the response time (the definition is based on the time duration to reach 70% of the full response of the sensor) of the gas sensors is short, as depicted in Figure 6c. However, if the definition is based on the time duration to reach 90% of the full response of the sensor, the response time is longer.

It is noted that both response and recovery times are delayed with the decrease in the concentration of the target gases. Desorption would be easier in an atmosphere with a low concentration of gas, and the recovery time should be shorter when the response time is prolonged. Furthermore, a faster recovery time is possibly related to the use of pumping, which could significantly accelerate the desorption process.

**Figure 7:**
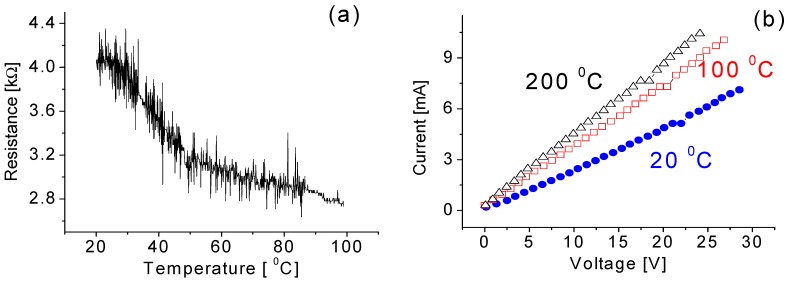
(**a**) Variation of resistance of the nanowires following the increase of temperature; and (**b**) the temperature effect on current-voltage curves.

The operating temperature greatly influences the properties of sensing nanowire materials. Therefore, before characterizing the temperature effect on the sensing behavior such as sensitivity and response time, the relationship between the resistance of the nanowires and the temperature was determined. Figure 7 shows the dependence of the resistance of the tungsten oxide nanowires as a function of temperature. Clearly, the resistance of the nanowires reduces from 4.1 kΩ to 2.8 kΩ with an increase of the temperature from 20 °C to 100 °C. The characterizations of electrical properties were also conducted at 20 °C, 100 °C, and 200 °C, as shown in Figure 7b. Two conclusions can be made based on the data in Figure 7b: (1) at a fixed temperature, the electrical current and voltage always have a linear relationship, indicating the ohm contact between the electrodes and the nanowires, and that there is no more charge accumulation or polarization effect in the circuit; and (2) the electric properties of the nanoparticle-based nanowires are sensitive to temperature. The reason of this is that zero-dimension structures like nanoparticles have much strong mutual link condition. Thus, the intrinsic structure of nanoparticles will undergo a great change with temperature.

**Figure 8:**
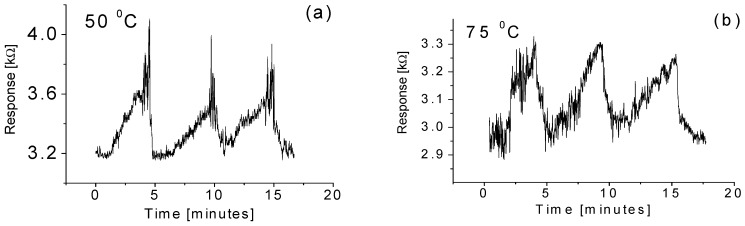
Responses of the sensor to the methane gas with a 2 ppm concentration at operating temperatures of (**a**) 50 °C; and (**b**) 75 °C.

Temperature effects on the responses of the sensor are depicted in Figure 8. When the sensor was exposed to the 2 ppm methane gas at an operating temperature of 50 °C, the output of the sensor increased and then reached a relatively stable value. When the inlet valve for the methane gas was switched off, the resistance abruptly decreased and then gradually reached a relatively stable value. Good reproducibility and short recovery time down to a few seconds have been obtained. However, no obvious improvement for the response time was observed. A similar phenomenon was also observed for the case at the operating temperature of 75 °C as shown in Figure 8b. A likely reason explaining why there is no outstanding rise time is the time needed for the gas to diffuse until reaching a balanced state inside the chamber.

It is important to point out that good baseline stability is always visible at any operating temperature, but the resistance/output of the response of the sensor is slightly affected after several exposures to the methane gas at high temperatures, as shown in Figure 8. A slight drop in the output (around approximately 1%) is observed when the sensor is exposed to the targeted gas every cycle. Such a drop is possibly due to chemisorption of molecules, which is responsible for changes in the electrical resistance of metal oxide at relative high temperatures due to the incomplete desorption of methane molecules on the surface of the nanowire sensor.

**Figure 9:**
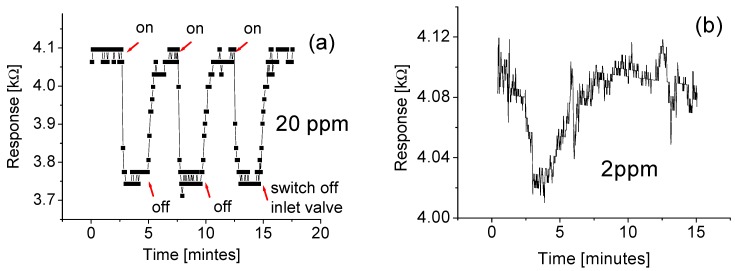
Responses of the sensor to the hydrogen gas at room temperature with the concentration of (**a**) 20 ppm; and (**b**) 2 ppm.

As a comparison, the fabricated sensor is also employed for detecting hydrogen gas. It was found that the response of the fabricated sensor to the hydrogen gas was completely different from that to the methane gas. Figure 9 shows responses at room temperature when the sensor is cycled between the “switch-on” and “switch-off” of exposure to the hydrogen gas with the gas concentration of 20 ppm and 2 ppm, respectively. Important stability or repeatability features obtained from the cycled test are clearly visible. The tungsten oxide composite-based gas sensor responded to 20 ppm hydrogen gas by decreasing its resistance, and then quickly reached a relatively stable value. The response time of around 5 s and the recovery time of around 10 s have been obtained.

When the hydrogen concentration decreases down to 2 ppm, the output of the signal strength drops almost 90% as shown in Figure 9b. Furthermore, both the rise time and fall time extend to around 1.5 min. This phenomenon is similar to that of the sensor exposure to the methane gas. The possible reason is that not enough molecules are adsorbed onto the surface of the nanowires for the reaction.

It is generally known that the gas-sensing mechanism is based on the interaction between the negatively charged oxygen adsorbed on the tungsten oxide surface and the targeted gas [26]. When the oxide semiconductor-based sensor is exposed to H_2_, hydrogen molecules react with these adsorbed atoms to increase the conductivity of the WO-based sensor by releasing electrons as shown in Figure 9. However, such an interpretation cannot be used to explain the response of the sensor to the methane gas as shown in Figure 5 and Figure 8.

It is noticed from the XRD spectrum of the synthesized nanowires in Figure 3b that there is a large amount of metal W component mixed with the WO material. If the sensor is exposed to the methane gas, these gas molecules would not only react with the negatively charged oxygen adsorbed on the tungsten oxide surface but also with the metal W component. Once the targeted gas molecules and the metal W dominated the reaction, it would result in reducing conductivity as shown in Figure 5. This change in the sensitivity could be attributed to a synergistic or competitive effect. Here it should be mentioned that analyses of the data obtained from additional experiments with higher concentrations of W indicated that a higher output/response of the fabricated sensor to methane gas has been obtained. In contrast, it does not significantly affect the sensor’s response to hydrogen gas. This evidence supports our explanation of the gas-sensing mechanism that when the targeted gas molecules and the metal tungsten dominated the reaction, it would result in the decrease of conductivity or an increase of its resistance.

## 5. Conclusions

We conclude that each as-grown wire mainly consists of a large amount of nanoparticles. The sample exhibits mixed states including WO_2_, WO_3_, and W. The metal W and poly-crystalline WO_3_ dominate the composition of the sample. Experimental data indicate that composite nanowires directly deposited onto a pair of electrodes can improve the performance of gas sensors such as good repeatability and stability. The response and recovery times of the fabricated sensors at 2 ppm concentration are about 1.5 min and 1 min, respectively. In contrast, it is only few s at the concentration of 10 ppm.

The operating temperature has a great influence on the properties of the sensor. A quick recovery time down to a few seconds has been observed, but there is no obvious improvement in response time. The reason why there is no outstanding rise time can be attributed to the time needed by the gas to diffuse itself until reaching a balanced state inside the chamber.

Differing from the response to the methane gas, the fabricated sensor responded to hydrogen gas by decreasing its resistance. Response and recovery time to the hydrogen gas are less than 5 s operated at 20 ppm, and 1.5 min at 2 ppm.

The general interpretation for the gas-sensing mechanism is based on the interaction between the negatively charged oxygen adsorbed on the surface of the composite and the targeted gas. Since there is a large amount of metal W component mixed with the WO material in the present case, if the sensor is exposed to the targeted gas, these gas molecules will not only react with the negatively charged oxygen adsorbed on the tungsten oxide surface, but they will also react with the metal W component. The change in the sensitivity is attributed to a synergistic or competitive effect.
